# Falls from Height in the Construction Industry: A Critical Review of the Scientific Literature

**DOI:** 10.3390/ijerph13070638

**Published:** 2016-06-28

**Authors:** Evan A. Nadhim, Carol Hon, Bo Xia, Ian Stewart, Dongping Fang

**Affiliations:** 1 School of Civil Engineering and Built Environment, Queensland University of Technology (QUT), Queensland 4001, Australia; carol.hon@qut.edu.au; 2 Institute of Health Biomedical Innovation, Queensland University of Technology (QUT), Queensland 4001, Australia; i.stewart@qut.edu.au; 3 Department of Construction Management, Tsinghua University, Beijing 100084, China; fangdp@tsinghua.edu.cn

**Keywords:** fall from height, construction accidents, construction activities and prevention strategies

## Abstract

Globally, falls from height (FFH) are a substantial public health jeopardy and are among the important leading causes of serious and fatal injuries for construction workers. A comprehensive understanding of the causal factors in FFH incidents is urgently required; however, the literature appears to lack a scientific review of FFH. In this study, 297 articles that contribute to the topic of fall incidents were reviewed. Seventy-five (75) articles met the criteria for relevance and were aggregated in a database to support a critical review. A synthesis of macro-variables approach was adopted rather than a structured meta-analysis. Such a method of analysis provides the flexibility to combine previous studies' findings. The most common factors associated with FFH are risky activities, individual characteristics, site conditions, organizational characteristics, agents (scaffolds/ladders) and weather conditions. The outcomes contributed to identifying the most significant research area for safety enhancement by improving engineering facilities, behaviour investigations and FFH prevention methods.

## 1. Introduction

The construction industry has grown over the last decades and resulted in improvements in company profits, financial accessibility and increased commodities demand in Australia [1], as it has in the United States [2] and in other countries. Despite its importance, it has long been identified as one of the most hazardous industries in many parts of the world [3]. Construction projects are described as dynamic and complex because of their sites and manpower. They are also characterised as temporary and transitory [4,5]. For example, the workforce for construction firms is usually temporary employees. Because construction crews spend most of their time at construction sites, they are exposed to a higher probability of injury or death [6]. Occupational accidents in the construction industry are frequent, and may lead to permanent disabilities and a high rate of fatalities [7].

Slipping, tripping and falling incidents in construction have received significant attention and have been widely researched due to their severity and frequency [8]. However, falls from height (FFH) still consistently have the highest rates amongst construction accidents compared with others types of accidents (e.g., vehicle collisions, hits by moving or falling objects, being trapped between stationary and moving objects and contact with electricity) and when compared to other industries’ accidents [6].

Of all construction accidents, falls are the leading cause of serious injuries (48%) and fatalities (30%) [9]. In particular, FFH represent more than one third of construction injuries, and are leading causes of multi-serious injuries and fatalities [10]. In 2013, FFH accounted for 36.9% of the occupational fatalities in the United States [11], 31% in the United Kingdom [12], and 12% in Australia. Various studies have collected data through different methods, including survey questionnaires, interviews, case studies, forensic and morbidity records and empirical/experimental laboratories to define the factors involved in falls. Yet the multidimensional relations among factors have not been investigated holistically. Previous review articles have concentrated on limited interventions, such as factors influencing activities, scaffolds/platforms or fall prevention solutions and field research of workers’ behaviour [9].

Construction fatalities and serious injuries caused by falls have attracted several researchers’ attention. For instance, Hu et al. [9] conducted an overview of factors influencing fall risks in general. Although it is useful to have such an overall view of factors, most of the reviewed papers were published prior to 2009, and some of them did not mainly focus on fall accidents. More recently, Jebelli et al. [13] have also conducted a study to validate the usefulness of postural stability metrics. Yet it is urgently required to address the major factors of FFH first, and then to invent or develop specific solutions to reduce/mitigate fall incidents.

Because there is a lack of studies that address fall factors, this is an appropriate time for a critical scientific review of the existing literature on FFH incidents and to look for further information for future research directions. It would be beneficial to have a comprehensive review of the literature, which could assist researchers and practitioners to grasp the body of knowledge and pave the way for future research to fill an important research gap. The present overview has covered several FFH facets, including data collection types, analysis methods, fall factors and prevention strategies. This review has provided an aggregated database that attempts to fill the knowledge gap.

## 2. Research Methodology

The literature was first searched to retrieve the relevant journal papers and conference proceedings from the accessible databases. As we are in the technological era, online databases were reviewed by utilizing the well-known search engine Google Scholar and the online library of the Queensland University of Technology to access the most common databases, including Science-Direct, Taylor & Francis, J-STOR, the American Society of Civil Engineering, Emerald Insight and many others that have published material on the topic of FFH incidents. As a starting point, a web search was conducted with typical keywords related to the subject, such as “fall(s)/falling from height(s)”,” fall(s)/falling from elevation”, “fall(s) injuries/accidents /incidents” and “construction industry/sector”. The search process resulted in accumulated knowledge through reviewing the collected literature. An additional number of keywords boosted and expanded the search process. They included “fall from roof/scaffolds/ladder/platform”, “fall to lower level/same level/opens”, “falls protection”, “hazardous/risky fall(s)”, “fatal fall(s)”, “and occupational accidents/injuries”.

After a comprehensive search on FFH publications, a relevancy selection criterion was adopted to select the closest study/paper to the topic. Papers that met the criteria of homogenous title, bibliographic keywords and abstracts were added to the database to be analysed by macro-variables. The review methodology towards the selected articles has taken two paths. Firstly, examining the title, keywords, and abstracts were checked to determine whether the papers were related or not. 297 publications related to FFH were found covering journal articles, preceding conference and reports. The second path was to check the references of the first path articles to reach the 75 articles to be synthesized in a database. The timeframe of the search for reviewed papers was about twenty years published from 1994 to 2014.

**Figure 1:**
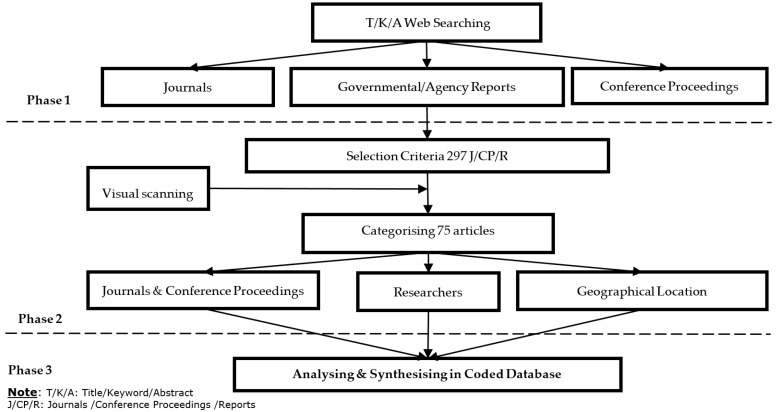
Research Methodology.

The 75 articles, which have been included with references, were comprehensively reviewed and synthesised into different categories to obtain the main variables that served the study aim. Figure 1 represents the three-phase methodology of this study. A total of 297 studies/reports were found by implementing the selection criterion. By narrowing down the topic and restricting these articles to research papers, 75 papers were finally selected for analysis.

## 3. Data Analysis

### 3.1. Selected Papers Profile

The data extracted from each article included the author, journal, title, keywords, study type, fall type, fall causes, fall effects, statistical /methods used, worker’s trade, activities performed, geographical location, building type, final results and conclusions (see Appendix A, Table A1). Table 1 shows the journals/conference proceedings and the numbers of papers reviewed.

**Figure 2:**
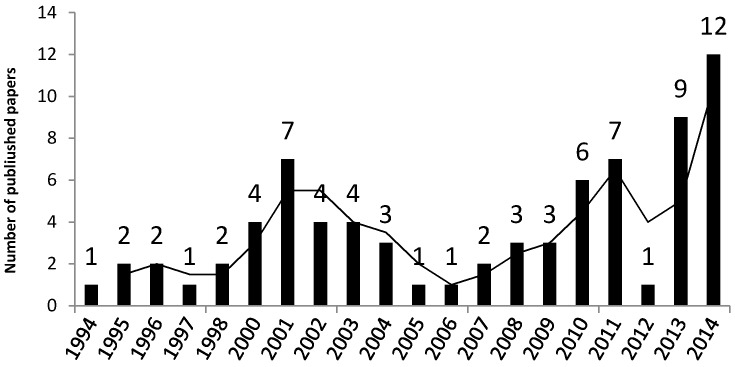
Numbers of studies by year.

As shown in Figure 2, there has been a clear fluctuation in the number of FFH papers in the last two decades. The increase in publishing in recent years is due to the large concern over the repetitive FFH accidents in the construction industry [14].

**Figure 3:**
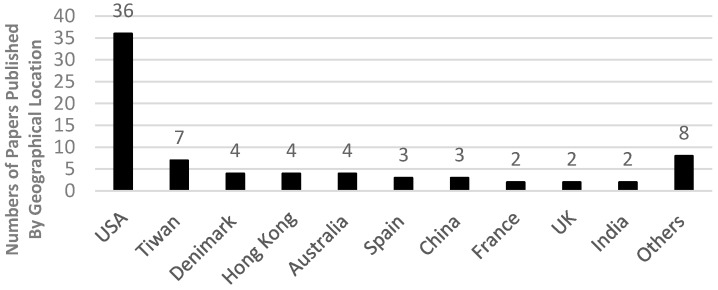
Numbers of studies by geographical location.

FFH studies were conducted in different countries, as shown in Figure 3. The highest ratio (48%) was from United States, followed by Taiwan (10%) and 5% each from Denmark, Hong Kong and Australia. Table 2 shows the sample sizes used by the 75 articles. Table 2 shows only the articles that collected and analysed data related to FFH; the rest discussed other FFH issues. More than half of the studies (54%) used a sample size of between 50 and 1000 cases, which reflects good statistical measurements. The period of data used by the 58 articles ranged from half a year to 20 years.

Table 3 shows the trades exposed to FFH. Around 20% of the trades were manual labours (skilled and unskilled workers) and masons, followed by carpenters and roofers, respectively. Among the different construction activities covered in the literature, those involving scaffolding and roofing were the most common, as shown in Table 4, which addresses the most frequent types of tasks/activities performed while FHH occurred. Both Table 3 and Table 4 identify the trades and activities most frequently related to FFH in the 75 articles.

FFH were analysed in different construction sectors, such as residential, non-residential, light/heavy commercial (including public buildings), and highway/road construction [15]. Table 5 shows the residential sector has a considerable portion (41%) of the analysed sectors.

### 3.2. Measurements Used

The methodological approaches implemented in the 75 articles were based on their research objectives. Data collection methods varied from one study to another, ranging from using archival data (demographic and fatalities), survey questionnaires, interviews, experiments and simulations, as shown in Table 6. Of all the papers, more than three quarters (76%) of the studies relied on archival data to understand the reasons behind FFH accidents.

Quantitative analysis approaches were mainly implemented to analyse and evaluate FFH incidents. Over 80% of the studies (65 articles) have taken only a statistical analysis approach. More than two thirds of the studies (76%) relied on archival datasets for the purpose of statistical estimations for evaluating and predicting accidents’ severity and fall patterns. Eleven percent of the studies used questionnaires/surveys and interviews.

**Figure 4:**
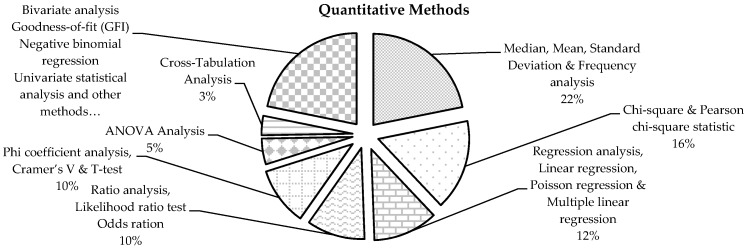
Quantitative approaches/methods used in the data analysis.

The studies conducted earlier tend to use autopsy records to analyse demographic data (e.g., age, gender, weight, etc.), fall locations and heights, and autopsy findings (e.g., injury severity, actual health conditions and causes of death). On the other side, the majority of the studies conducted their analysis by investigating the interventions of fall factors (e.g., height, occupation, personal protective equipment (PPE), weather and workers’ safety behaviours and attitudes) [9] or specific work-related fall issues, such as workplace conditions, training requirements and curricula for avoiding future FFH accidents [16,17]. More than two thirds of the studies 54 utilised statistical methods to evaluate FFH incidents in the construction sector, while the rest of the studies discussed factors/reasons. Table 7 shows the statistical methods used in more detail, and Figure 4 presents the statistical methods used.

Quantitative analysis approaches were mainly implemented to analyse and evaluate FFH incidents. Over 80% of the studies (65 articles) have taken only a statistical analysis approach. More than two thirds of the studies (76%) relied on archival datasets for the purpose of statistical estimations for evaluating and predicting accidents’ severity and fall patterns. Eleven percent of the studies used questionnaires/surveys and interviews.

The studies conducted earlier tend to use autopsy records to analyse demographic data (e.g., age, gender, weight, etc.), fall locations and heights, and autopsy findings (e.g., injury severity, actual health conditions and causes of death). On the other side, the majority of the studies conducted their analysis by investigating the interventions of fall factors (e.g., height, occupation, personal protective equipment (PPE), weather and workers’ safety behaviours and attitudes) [9] or specific work-related fall issues, such as workplace conditions, training requirements and curricula for avoiding future FFH accidents [16,17]. More than two thirds of the studies fifty-four (54) utilised statistical methods to evaluate FFH incidents in the construction sector, while the rest of the studies discussed factors/reasons. Table 7 shows the statistical methods used in more detail, and Figure 4 presents the statistical methods used.

### 3.3. Understanding FFH Accidents

FFH is at the forefront of construction industry incidents compared with other industries [6]. Yet investigating how and why FFH accidents happen has still not received a sufficient amount of attention. To understand this, researchers should investigate accident causation and human error theories [46]. A number of studies in the literature have investigated in depth the factors/reasons that might influence FFH accidents in terms of proportions, rates and relationships [13,47]. The factors present in FFH represent the circumstances or impacts that contribute to fall accidents. There are several categories of generic factors that have been studied, such as individual behaviour, the construction surroundings, safety regulations/standards, task/activity performed, platforms/surfaces, and environmental conditions [3,9]. These factors are interrelated with each other. For instance, worker experience affects individual status, and company policy has an impact on training courses and shift-work timing [4].

Table 8 represents the types of positions that can be categorised as falling from agents, surfaces and buildings. Fall from agents includes fall from scaffolds/ladders [10,18,23,48]. Fall from surfaces contains falls from platforms, openings, walkways, skylights, and other frequently used objects [9,26]. Falls from buildings is the most hazardous when working at height due to the overlapping factors involved in falling (e.g., wind velocity, structure height, risky activities, workers’ attitudes) [4,26].

FFH incidents have adverse physical effects. The most common injuries are fractures, trauma, contusions, concussions, bruises and abrasions. They may lead to death. After the physical injury which has had a direct influence on a worker’s body, in some cases, a psychiatric illness may also occur due to the loss of a job opportunity or from having a permanent disability [19]. Table 9 lists the major effects of FFH incidents according to the studies.

### 3.4. Leading Factors to FFH Accidents

FFH are influenced by a number of factors, including risky activities, individual characteristics, site conditions, organisation/management, agent, and weather/environmental conditions. Table 10 lists the factors and variables leading to FFH accidents. The two most frequently mentioned causes of FFH are: (1) risky activities (trades requiring working at height); and (2) individual characteristics (demography, knowledge level and human behaviours).

#### 3.4.1. Risky Construction Activities

FFH injuries are more likely to occur when the construction crew performs hazardous tasks/activities [55]. The riskiness of the construction occupations varied among the 75 articles. Scaffolding and roofing, in Table 4 for instance, are the most dangerous construction that lead to FFH. Roofing workers are susceptible to fall-related hazards because of fragile roofing material and handling heavy tools or equipment [27,56]. In some cases, task complexity/hardship diverting workers’ attention while at significant heights could be a leading cause of FFH [57].

#### 3.4.2. Individual Characteristics

Individual variables play a significant role in FFH accidents. The responsibility for falling is partially situated in the individual characteristics of the construction workers, including their demography (e.g., age, gender, and weight), knowledge level (education and experience), human behaviours and attitudes, physical characteristics, and health (e.g., chronic disease and fatigue). At the top of the list of characteristics is the workers’ demography. Demographic attributes such as age, gender, weight, marital status, etc., have strong relationships with the workers' health, education and experience and nutrition system. For instance, workers who are suffering from obesity or who are overweight are exposed to fatigue more often, and fatigue is an important leading factor to FFH [47]. Older workers are exposed to FFH more than younger ones [55]. The second is knowledge level: Construction crews are more likely to experience FFH if they lack education, skills, dexterity and experience [9,15,69], ignore attending safety training courses [16,24,70], and have poor work practice, capabilities, communications and tolerance (e.g., [18,32]. The third and the most important characteristic is human behaviour. The workers’ behaviour (e.g., carelessness, stupidity, misjudgement or overconfidence) is one of the major causes of permanent disability or death after falling from height [69]. Such behaviours threaten the workers’ lives, regardless of their experience, education level or position. The fourth attribute is sleep deprivation and work depression. There is a clear correlation between workers’ health and fall hazards; this is caused by a chain of factors, such as workload and pressure, and sleepiness or sleep deprivation [47,71]. Finally, fatigue has been defined as a major risk factor for shift workers [81]. It comes from chronic work pressure or burnout [82] and is generated from increased workloads [57] with intensive physical effort or overexertion for usual or unusual actions [6,71]. Fatigue is influenced mostly by the workers’ health status and physical characteristics. Moreover, working at height for long intervals could increase the susceptibility to fatigue because of work posture such as changes in platform/surface properties [72] or because of the need to increase awareness and consciousness [6,48].

#### 3.4.3. Site Conditions

In one hand, platform/surface conditions can impact those who work at elevated heights in several ways. FFH could occur when there are defects in the work surface, such as unprotected walkways, improper guardrails, slippery or sloped surfaces. Unexpected modifications in surface properties can be sufficient for FFH to occur [43,49]. On the other hand, construction sites are sometimes operating for twenty-four hours. Insufficient lighting/illumination on night-shifts can affect for the visibility of the surroundings. As a result, workers might fall from high surfaces due to a lack of illumination [70].

#### 3.4.4. Organisation/Management

Organisation’s variables are significantly associated with FFH accidents and it has several components that lead to FFH. Firstly, the Australian construction companies are often small businesses, and 97% of general construction businesses employ 20 workers or less. Given that more workers are increasingly involved in small businesses due to short employment periods and high wages [3,28,69]. Company size has a strong negative relationship with the incidence of FFH accidents [5]. Small companies might have improper safety measures/standards, such as insufficient/inoperative personal protective equipment (PPE), personal fall arrest systems (PFAS), defective safety belts/harnesses, and lack of training courses offered, especially for safety [16,24,41,70]. Riskier work is usually conducted by small to medium sized companies rather than by large companies due to job distributions and time planning/economizing [69].

The second component is contractors and sub-contractors. A lack of capability or of resources of contractor/sub-contractors also leads to FFH. Although contractors and subcontractors have more direct control in construction sites, they need to implement a complicated system to process the safety regulations on a day-to-day basis [58]. FFHs can be significantly avoided by personal protective equipment (PPE) and personal fall arrest systems (PFAS). However, inadequate or inoperative safety equipment, improper use of this equipment, and non-availability of protective resources and procedures will lead to FFH [6,41,69,73,78].

The third component is project management. Project timelines or shift work generate pressure on workers’ attitudes/behaviours to complete on time or ahead of schedule because of the urgency or supervisors’ pressure to accelerate the tasks with incompatible safety standards [59], which makes FFH injuries occur mostly in the afternoon hours [27].

#### 3.4.5. Agents

Agents are one of the influential variables, and were mentioned in more than 25% of the studies (e.g., [10,18,23]). Scaffolds and ladders are used to perform construction tasks at height. Primarily, positioning scaffolds and ladders in a risky manner might cause construction worker fatalities. Scaffolds can be very dangerous when they are improperly used or erected [6,73]. Furthermore, prolonged construction activities on a scaffold or ladder with an unreliable design have adequately explained the high rates of FFH [18].

#### 3.4.6. Weather/Environmental Conditions

The weather and surrounded environment in some cases have contributed to FFH incidents. Regionally, construction workers face heat, cold, rain or windy weather. The trades most exposed to weather conditions are carpenters and roofers, as shown in Table 3 [19,29,80]. It is not possible to change the weather, so the workers’ behaviours need modifications based on the weather [16].

### 3.5. FFH Prevention Strategies

The significance of studying fall accidents is to prevent and mitigate the seriousness of injuries. Around half of the articles (51%) have given either recommendations or preventive methods to avoid falling incidents. These articles have been divided into passive or proactive methods. 10% of the articles have provided precautionary/proactive measures inside construction sites. The most important protective methods are to have on-site precautionary measures, and education and training are at the forefront of preventing FFH accidents, while the importance of passive methods is sometimes lies on analysing the fall accidents data for future plans.

Proactive methods can be the optimal strategy to reduce FFH incidents. Educating and training construction workers can also reduce serious incidents. Furthermore, designing short safety training courses for the workers, seminars and talks focusing on work at height risks, might have positive impacts on workers’ behaviour that reduce FFH incidents [28,70]. Further, minimizing the amount of hazardous agents and the duration of exposure can mitigate or reduce the severity of FFH [3,60]. Moreover, researching and restudying to improve unsafe designs could affect positivity on FFH injuries' reduction. For instance, there is a need to redesign scaffolds to reduce their complexity and so that it takes less effort to erect and dismantle them, allowing them to be used reliably to reduce their associated risks [14].

Majority of industries, including the construction industry, are subjected to specific safety standards/guidelines. The importance of such regulations is to enhance the on-site health and safety management to prevent (falling) injuries while working. Regular safety regulations revisions and regular inspections are among the potential remedies that can include any variation in job design, for example, weather or platform conditions [74]. Table 11 summarises the recommendations and methods to reduce or prevent work-related FFH.

## 4. Discussion

This paper conducted a comprehensive review of FFH literature. It contributes to summarising the research trend of FFH, highlighting the inadequacies, and providing signposts for future research. The review shows that most of the existing FFH research focused on analysing archival data. Sources of the archival data are mainly accident databases or accident reports. There are limitations of using accident databases or accident reports which are secondary data for analysis.

Accident databases may not have been designed in a way to fit the purpose of the FFH research. Categorization of the data in the accident databases may not align with the needs of the researchers conducting FFH research. This data limitation restrains FFH research from providing further in-depth analysis.

The problem of solely using accident reports as the data for FFH research is that the research has the problem of hindsight bias [84]. As explained by Dekker [84], hindsight means “being able to look back, from the outside, on a sequence of events that led to an outcome that has already happened”. According to Dekker and Hofmeyr [85], hindsight bias permeates in almost every accident report. After the accident has happened, it is easy to point out what went wrong and what action should have been taken. It is impossible for the investigator to go back to the past to fully understand the world that someone faced who did not have the knowledge of the outcome of the action. The action taken might be the most appropriate action to have been taken when not knowing the outcome of the action. It is not uncommon to find in the accident reports the root cause of accidents and what should have been done or not been done. However, this may not truly reflect the situation before the accident happened.

There is no doubt that learning from incident is important. However, most of the extant FFH literature did not embrace learning theories and methods. This limits the potential of the FFH literature to provide theoretically supported recommendations and strategies for organizations to develop the learning capability to prevent FFH accidents. Learning from accident investigation is an emerging topic of safety research. To effectively learn from FFH incident, interdisciplinary learning science theories and research methods should be embraced. For example, learning from incidents involves a number of critical steps, namely investigating and analysing incidents, planning interventions, intervening, and evaluating [86]. These steps should be considered when conducting FFH research. Adopting some learning from incident models for FFH research would be useful as well. For example, Lindberg, Hansson and Rollenhagen [87] developed a CHAIN model for lesson learnt dissemination and promulgation of preventive actions.

A vast majority of the existing FFH research studies did not consider the holistic systems approach, that is, how the factors are interdependent with one and other. As evidenced in the review, individual factors were the most frequently mentioned. Interdependencies of the factors are underexplored in the existing literature. Factors leading to FFH accidents at the blunt end are likely to be undermined but the factors at the sharp end tend to be overestimated. In an increasingly complex socio-technical environment, it is unlikely that accidents have a single root cause or follow a sequence event pattern [85]. Rather, safety is an emergent property arising from the interaction of systems [88]. Future FFH research should take a systems thinking approach to reveal the interdependencies of factors [88]. Systems thinking approach will enable more rigorous statistical analysis to be conducted, such as system dynamics modelling and Bayesian networks. Revealing the interdependencies of the FFH accidents will be useful in formulating more effective and realistic preventive measures.

To further advance FFH research and make an impact, it is suggested that other sources of data and research methods should be employed. Cognitive mapping for interviews and critical incidents can be useful in extracting the details of how FFH accidents happened without oversimplifying the complexities or losing important details [89]. Getting a diversified view of safety experts, industry practitioners and even workers experienced FFH non-fatal incidents would probably be useful in deriving a balanced view on how to prevent FFH accidents. Scientific experimental studies have the merit of drawing causal relationship. Utilizing experimental research design with behavioural or physiological parameters on FFH research can substantially advance the existing knowledge.

Although design for safety has been one of the common recommendations found in the existing FFH research, this concept has yet to be adopted from the whole lifecycle perspective. Design for safety has huge potential to design out or minimize the FFH risk not only during construction but also long-term repair and maintenance of the building or infrastructure. Future FFH research should put more emphasis on the intervention of technology, such as building information modelling (BIM), augmented virtual reality, wearable monitoring devices for safety and health. Recently, there are studies [71,90] which utilise smartphones to detect fall portents for construction workers. BIM has been utilized for automatic safety checking of construction models and schedules [91] and fall hazard identification [92]. Scaffolding and roofing have been identified as the main activities leading to FFH accidents. Further research can be done to improve the design of scaffolds. For example, Collins et al. [93] has tried to integrate safety risk factors in BIM for scaffolding construction. Further research can be done along this line. Similar research is also worth conducting with temporary formworks and roofing. Prefabrication is another important direction that should not be neglected. Prefabrication has the advantage of reducing the time for workers to work at height. Currently, its benefit to reduce risks of FFH is often inferred and empirical research evidence for the improvement has not been well-documented. There are potentials for future FFH research to determine the impacts of adopting prefabrication on reduction of FFH risks and improvement of safety performance.

## 5. Conclusions

To conclude, a critical review has been undertaken to summarise the existing FFH literature, highlight the inadequacies and provide future research directions. The reviewed FFH literature was mainly drawn from peer-reviewed journal articles and conference proceedings [3,4,5,6,7,9,10,13,14,15,16,17,18,19,20,21,22,23,24,25,26,27,28,29,30,31,32,33,34,35,36,37,38,39,40,41,42,43,44,45,47,48,49,50,51,52,53,54,55,56,57,58,59,60,61,62,63,64,65,66,67,68,69,70,71,72,73,74,75,76,77,78,79,80,83] from the available databases. It was found that most FFH literature utilizes archival data as the primary source of data analysis. Exiting FFH literature lacks theoretical-base of learning theories and methods. To further advance the knowledge, it is suggested that more innovative research methods and other source of data should be utilised for future FFH research. Future FFH research will likely need to be more interdisciplinary, for example combining the knowledge of information technology, behavioural experiment, systems thinking, and ergonomics to derive a holistic approach to tackle FFH risks. Findings and recommendations of this study provide insightful signposts for safety researchers to conduct future research and shed some lights for industry practitioners to design preventive measures for FFH accidents.

## Figures and Tables

**Table 1:** Journals and conferences proceedings papers reviewed.

No.	Journals	Articles Met Criteria
1	*Journal of Safety Research*	10
2	*Safety Science*	7
3	*Ergonomics*	5
4	*Accident Analysis and Prevention*	5
5	*Journal of Construction Engineering and Management.*	4
6	*Journal of Occupational and Environmental Medicine*	4
7	*Research and Practice for Fall Injury Control in the Workplace (conference proceeding 2010)*	4
8	*Construction Management and Economics*	3
9	*Forensic science international*	3
10	*American Journal of Industrial Medicine*	3
11	*Computers & Industrial Engineering*	2
12	*International Journal of Industrial Ergonomics*	2
13	Other Journals (less than 2 papers per journal)	22
	**Total**	75

**Table 2:** Summary of sample sizes used in selected papers.

Sample Size	No. of Articles	Percentage
0<	3	4%
50≤	19	25%
500≤	7	9%
1000≤	15	20%
5000≤	5	7%
10,000≤	5	7%
50,000≤	3	4%
100,000≥	1	1%
Total	58	77%

**Table 3:** Trades exposed to FFH.

Worker Trade	No. Articles
Manual labour (Skilled & unskilled workers) & masonry	26
Carpenter	17
Roofer	14
Electrician	10
Painter	8
Plumber & pipefitter	8
Ironworker	7
Drywall	4
Scaffolder	4
Sheet/Structural metal installer	3
Insulation installer	3
Forklift operator	1

**Table 4:** Construction activities lead to FFH.

Performed Activity	No. Articles
Erecting/dismantling of scaffold	20
Roofing	18
Painting	10
Plumbing	7
Drywall/Wall covering	6
Electric tasks	6
Carpentry	6
Installing sheet/structural metal installer	4
Precast Concrete	3

**Table 5:** FFH studies within different construction sectors.

Construction Type	No. Articles	Percentage
Not mentioned	36	48%
Residential	31	41%
Commercial (light& heavy commercial & non-residential & public buildings)	16	21%
Highway/Road	8	11%

**Table 6:** Data collection strategies.

No.	Data Collection Type	No. of Articles	Percentage
1	Archival data	57	76%
2	Questionnaire/Survey/Interview	8	11%
3	Experimental data	5	7%
4	Simulated data	1	1%
5	No FFH accident data used	4	5%
**Total**		75	100%

**Table 7:** Statistical methods used on FFH papers.

Statistical Method	Purpose	No. Articles	References
Median, Mean, & Standard Deviation	Simple Statistical Description	6	[10,18,19,20,21,22]
Frequency analysis	Simple Statistical Description	4	[14,23,24,25]
Chi-square & Pearson	Statistical relationship significance analysing, comparison, computing differences & responses among factors, studying variables relationships and correlations analysis	14	[3,4,10,19,23,26,27,28,29,30,31,32,33,34]
Regression analysis, Linear regression, Poisson regression & Multiple linear regression	Examine for statistically significant, calculate adjusted rate ratios, determine significant predictors, and determine amount of variance	10	[5,29,31,35,36,37,38,39,40,41]
Ratio analysis, Likelihood ratio test & Odds ration	Assess relative hazards, Extrapolate among factors and Compare population groups.	6	[4,5,17,26,27,36]
ANOVA Analysis	Analysis of variance, determine causes, identify significant differences, compare performance in different groups, account for intervention/control status, and determine relationships.	4	[19,32,37,42]
Cross-Tabulation Analysis	Determine factor relationships, provide more specific injury descriptions.	3	[26,30,43]
Phi coefficient analysis & Cramer’s V, *T*-test	Examine relationship among factors.	7	[3,27,30,33,36,37,44]
Other methods	For significance of statistical modelling and sufficient representing, Investigate overlapped relationships, etc.	10	[3,10,26,28,30,32,38,40,41,45]

**Table 8:** FFH categories.

Fall from Height Categories	Expression Used	Proportion of Appearance
Agents	Fall from scaffold	23%
	Fall from ladder	17%
Surfaces	Platform, openings, walkways & skylights and other high objects	11%
Buildings	Fall from roof/building	16%
	Fall from (same/different/lower) level	15%

**Table 9:** Most common FFH Effects.

No.	Fall Effect	No. of Articles	References
1	Mental/psychiatric illness	1	[19]
2	Fractures	11	[4,5,9,24,37,39,43,48,49,50,51]
3	Trauma	11	[4,5,9,19,26,34,37,39,49,52,53]
4	Contusion, Concussion, Bruise & Abrasion	9	[16,24,37,39,43,48,50,51,54]

**Table 10:** Leading Factors for FFH accidents.

No.	Factors	Variables	No. of Papers	References
1	Risky Activities	Working at Height: with complexity, hardship, prolong tasks	39	[3,4,5,6,7,10,14,15,16,18,21,24,26,27,28,30,31,32,35,36,38,39,42,44,47,48,49,50,57,58,59,60,61,62,63,64,65,66,67,68]
2	Individual Characteristics	Demography: age, gender, weight, ethnicity etc. Knowledge Level: lack of education, experience, training, etc. Human behaviours: misjudgement, attitude, unsafe behaviour & carelessness, etc. Workers health/characteristics: fatigue, sleep deprivation or depression	31	[4,6,7,9,15,16,19,24,26,27,28,30,31,33,38,40,45,47,54,55,57,66,69,70,71,72,73,74,75,76,77]
3	Site Conditions	Insufficient lighting & illumination Unprotected/defective platform & surface	13	[7,15,23,26,43,49,50,55,57,59,70,71,72]
4	Organization/Management	Small-medium sized companies: lack training programs; Contractors & sub-contractors: lack of proper/safe equipment; Shift work: night shifts and break periods; Project Timeline: pressure to accelerate.	11	[3,6,28,41,55,58,59,69,73,76,78]
5	Agent	Improper position or defective: ladder/scaffold (erecting/dismantling)	5	[6,16,23,73,79]
6	Weather/Environmental Conditions	Frost, snow, heavy rain, humid extreme temperatures, noise, dust, etc.	4	[19,29,41,80]

**Table 11:** Methods to reduce/prevent work-related FFH.

No.	Safety Strategies	Actions Taken to Prevent/Mitigate FFH Accidents	References
1	On-site Precautionary measures	Minimize amount of hazardous agents & duration. Professionally set up for agents (scaffold/ladder); Utilise technological agent: safety monitoring systems, positioning device systems, Controlled access zones, warning line systems. Site proactive actions: Guardrails, surface protections; Safety nets; Helmets; personal fall arrest systems (FAS); personal protection equipment (PPE); and Safety harnesses; etc.	[3,6,10,24,26,27,60,83]
2	Educating and Training	Design specific courses; Training for unskilled workers; Courses on how to use scaffolding agents (scaffold/ladder) properly; Workshops on safety for unskilled/new workers through safety seminars and talks; etc. Stimulate employees to follow safety regulations.	[27,28,58,70]
3	Safety Regulations	Frequent revision of safety regulations and regular inspections of sites.	[69,74]
4	Research and Development	Searching on-site potential risks related to FFH accidents; Systematic research on the behaviour of individuals & groups and construction companies.	[42,76]
5	Job redesign	Redesign weight for lifting (e.g., blocks and reduced-weight cement bags), Developing scaffold erection and dismantling methods, and Improve the ergonomics of the workplace (e.g., comfortable temperature, modest humidity, enough illumination level, noise reduction) to reducing the impact of FFHs.	[14]
6	Health protections	Mitigate height operations when workers suffer from physical disorders: Excessive fatigue; Sleepiness; Depression; etc. Shorten the periods of workers suffering from chronic disease: Hypertension; Heart disease; Anaemia; and Epilepsy; etc. Forbidding working at height if alcohol/drug has been detected.	[74]
7	Safety promotion	Roving exhibitions, banners and posters, Safety and health messages, Broadcasting dramas of documentaries through; Television, radio and mobile media; Articles/newspapers; etc.	[58]

## Appendix A

**Table A1:** An example of 2 records in the coded database.

No.	Analysed Categories	Paper 1	Paper 2
1	References	[26]	[27]
2	Journal	*International Journal of Construction Education and Research*	*Journal of Safety Research*
3	Title	Investigation of Factors Contributing to Fatal and Nonfatal Roofer Fall Accidents	Construction workers’ falls through roofs: Fatal versus serious injuries
4	Keywords	project management, quantitative research, roofer falls, safety management	Occupation; Risk factor; Falls from heights; Personal protective equipment; and Descriptive analysis
5	Study type	Analytical study	Analytical study
6	Fall type	fall from elevation	Fall from roofs
7	fall causes	1-unguarded /improperly secured platforms, etc. 2-Roofers can fall from roof edges, roof openings, etc. 3-fall factors: victim’s age, experience, trade, accident proximal and root cause, fall location, fall distance, injury characteristics, and site conditions,	1-type of elevation fall (e.g., fall from, fall through); 2-working surface (e.g., ladder, scaffold, roof); 3-construction branch and occupation; and 4-mechanism of the incident (e.g., roof tiles, roof sheets, and skylights/roof lights);
8	Fall effects	1-suffer trauma, 2-loss of life and 3-lowered productivity due to work-related accidents.	1-amputations, 2-bone fractures, and 3-injury to extensive parts of the body
9	Statistical/methods used	1-cross-tabulation analysis; 2-logistic regression modelling; 3-Univariate statistical analysis; 4-Pearson chi-square statistic; and 5-Odds ratio (OR)	1-Odds ratio, 2-chi-square test, 3-T test,
10	Worker’s trade	Roofer	carpenter, electrician, nonskilled
11	Activities performed	Roofing, skylights, and scaffolding	replacing roof sheets and installing insulation,
12	Geographical location	USA	Denmark
13	Building type	Residential	General Construction
14	Final results	Roofers most frequently experienced falls while working heights below 20 feet. Fall accidents involving roofers more frequently result in nonfatal injuries than in fatalities. Non-union roofers experience a higher frequency of falls than union roofers. Fall distances between 10 and 20 feet showed the highest fatality as well as nonfatal injury frequencies.	1-N = 38, 19 fatal and 19 serious injury fall incidents, majority of their working hours are before 1:00 p.m. 2-From 7:00 a.m. to 12:59, 8 incidents occurred in the morning hours and 2 incidents in the afternoon hours, 3-Eyewitness and autopsy reports, involved a worker suffering an alcohol-related abstinence seizure while working with a colleague on a roof.
15	Conclusions	1-This is attributed to the fact that roofers are specialized skilled trades; they are often not engaged in non-routine tasks. 2-the union status variable is not significantly related to the degree of injury. 3-Human factors involving misjudgement of hazardous situation and inappropriate choice of equipment/process produced the highest frequencies of fatal and nonfatal falls, where fatalities were fewer in both cases.	1-Majority of fatal injury falls occurred in the afternoon hours. 2-The majority of known times of fatal injury elevation falls in all economic sectors (42%), as well as in construction (42%), and the 10 cases focused on in this study (50%)—occurred in the 3-h time period between 1:00 p.m. and 3:59 p.m. 3-Occupational injuries in construction industries in different countries have identified roofing and roofers in particular, as having elevated risks of fatal falls from heights.
